# Determinants of breast cancer screening among women of reproductive age in sub-Saharan Africa: A multilevel analysis

**DOI:** 10.1371/journal.pone.0312831

**Published:** 2024-12-27

**Authors:** Beminate Lemma Seifu, Yohannes Mekuria Negussie, Angwach Abrham Asnake, Fraol Daba Chinkey, Bezawit Melak Fente, Zufan Alamrie Asmare

**Affiliations:** 1 Department of Public Health, College of Medicine and Health Sciences, Samara University, Samara, Ethiopia; 2 Department of Medicine, Adama General Hospital and Medical College, Adama, Ethiopia; 3 Department of Epidemiology and Biostatistics, School of Public Health, College of Medicine and Health Sciences, Wolaita Sodo University, Wolaita Sodo, Ethiopia; 4 Bin Haider Healthcare Center, Dubai, United Arab Emirates; 5 Department of General Midwifery, School of Midwifery, College of Medicine & Health Sciences, University of Gondar, Gondar, Ethiopia; 6 Department of Ophthalmology, School of Medicine and Health Science, Debre Tabor University, Debre Tabor, Ethiopia; Nofer Institute of Occupational Medicine, POLAND

## Abstract

**Background:**

Breast cancer is a significant global health issue, responsible for a large number of female cancer deaths. Early detection through breast cancer screening is crucial in reducing mortality rates. However, regions such as Sub-Saharan Africa (SSA) face challenges in identifying breast cancer early, resulting in higher mortality rates and a lower quality of life. Yet, there is a noticeable gap in the literature concerning breast cancer screening. Thus, this study aimed to estimate the pooled prevalence of breast cancer screening and associated factors among women of reproductive age in SSA.

**Methods:**

A weighted sample of 80,058 reproductive-age women from recent Demographic and Health Surveys in SSA countries was considered for analysis. A multilevel modified Poisson regression model with robust variance was fitted to identify factors associated with breast cancer screening. Four nested models were fitted, and the model with the lowest deviance value was selected. An adjusted prevalence ratio with the corresponding 95% confidence interval was used to measure the strength of the association. Finally, statistical significance was declared at a p-value < 0.05.

**Result:**

The pooled prevalence of breast cancer screening among reproductive-age women in SSA was 11.35% (95% CI: 11.14%, 11.56%), with variations ranging from 4.95% (95% CI: 4.61%, 5.30%) in Tanzania to 24.70% (95% CI: 24.06%, 25.33%) in Burkina Faso. Age (20–24, 25–29, 30–34, 35–39, 40–44, and 45–49 years), secondary and higher education, wealth index, media exposure, parity, contraceptive use, pregnancy status, breastfeeding status, and visiting a healthcare facility in the last 12 months were identified as significant positive determinants of breast cancer screening. Conversely, being a rural resident and having a primary education level were found to be negative determinants.

**Conclusion:**

This study uncovers a low prevalence of breast cancer screening in SSA countries, despite high associated mortality rates. Emphasizing the significance of targeted interventions, it highlights the crucial need to promote education and awareness regarding the benefits of breast cancer screening, particularly in light of the challenges faced by many women in the region.

## Introduction

Breast cancer is a common and significant global health issue, ranking among the leading causes of cancer-related deaths in women, contributing to 15.5% of all cancer fatalities, or 1 in 6 cancer deaths [1–3]. In 2020, 2.3 million women were diagnosed with breast cancer, leading to 30% of them dying from the disease. By 2040, the number of cases is projected to reach 3 million, with the death rate increasing to 33% [3, 4]. Low and middle-income countries (LMICs) bear a threefold burden of breast cancer, with Africa contributing 8.3% of cases and 12.5% of global deaths [3, 5, 6]. Although the overall incidence is lower compared to high-income countries, Sub-Saharan Africa (SSA) experiences disproportionately high mortality rates, and the incidence is projected to double by 2040, posing a serious public health challenge [3, 7].

Breast cancer is more common in later life but can occur at any stage after puberty [4]. Breast cancer screening is important for early detection of the disease which plays a crucial role in preventing, and potentially reducing mortality [8, 9]. Screening methods, including mammography (MMG), breast ultrasonography, breast self-examination (BSE), clinical breast examination (CBE), and magnetic resonance imaging (MRI), may be useful for reducing death due to breast cancer. While MMG is globally recommended as a standard screening test, CBE and BSE are also suggested for low-resource settings due to their cost-effectiveness [4, 10, 11]. Most Guidelines suggest yearly or biannual mammographic screening for women aged 40 to 74, with increased frequency for high-risk groups [12].

The economic and health landscapes have been adversely affected by the increasingly prevalent phenomenon of late-stage presentation of breast cancer [6, 13]. In LMICs, the majority of women often seek medical attention in the late stages of cancer [14]. The gravity of breast cancer lies in its lethality when not promptly identified and treated. Advanced stage and large tumor size upon diagnosis coincide with a lower chance of survival. LMICs, including SSA, grapple with a significantly low five-year survival rate, often hovering at or below 50% [6, 15, 16]. The delayed detection of breast cancer in these regions contributes to elevated mortality rates and a notable reduction in the overall quality of life in SSA [17, 18].

However, a discernible gap exists in the literature concerning breast cancer screening. Methodologically, previous studies in this domain have not utilized multilevel modeling to address the hierarchical structure inherent in the demographic and health survey (DHS) data [17, 19]. DHS are nationally-representative household surveys that provide data for a wide range of monitoring and impact evaluation indicators in the areas of population, health, and nutrition, covering regions in Africa, Asia, Latin America/Caribbean and Eastern Europe. Furthermore, these studies have not incorporated appropriate weighting techniques to compensate for the non-proportional allocation of sample sizes and ensure accurate estimation of standard errors. These methodological enhancements not only distinguish our study but also contribute to a more robust analysis of breast cancer screening and its associated factors in the SSA context. So, this study aids in understanding regional and country-specific variations in breast cancer screening, crucial for prioritizing interventions in high-risk countries. This nuanced knowledge empowers nations to address low screening rates through targeted, contextually informed approaches, maximizing the impact of interventions in SSA’s unique healthcare landscape.

## Methods

### Data source, study setting, and population

The most recent DHS data from six SSA nations were used in this study. DHS is a nationally representative survey routinely conducted every five years and gathers data regarding basic health parameters such as mortality, morbidity, fertility, and maternal and child health-related characteristics. The survey used a two-stage stratified sampling technique to select the study participants. In the first stage, Enumeration Areas (EAs) were randomly selected based on the country’s recent population, and using the housing census as a sampling frame, households were randomly selected in the second stage. Men, women, children, birth, and household datasets are all included in each country’s survey. The women’s questionnaire collects detailed information from women of reproductive age (usually 15–49 years) on topics such as fertility, family planning, maternal and child health, HIV/AIDS, gender-based violence, and other health-related topics through face-to-face interviews. Since the study population was reproductive-age women, thus, we used the individual (women’s) Record dataset (IR file). In the current study, 80,058 women of reproductive age were considered for final analysis. Detailed information about DHS methodology can be found in the official database https://dhsprogram.com/Methodology/index.cfm.

### Study variables and measurements

#### Dependent variable

Women were asked if a doctor or other healthcare provider examined their breasts to check for cancer. The examination could include either a clinical breast exam, in which a healthcare provider uses their hands to feel for lumps, or other changes, or the use of medical equipment to make an image of the breast tissue, such as a mammogram. The response was captured as “no  =  0” and “yes  =  1”.

#### Independent variables

Two levels of independent variables were taken into consideration, in keeping with the goal of the study and taking into account the hierarchical nature of the DHS data. At level-1 contained individual-level variables such as age, marital status, educational status, wealth status, sex of household head, media exposure, parity, pregnancy status, breastfeeding status, contraceptive use, and visited health facilities in the last 12 months. At level two, the community-level variables considered in this study were distance to a health facility and residence.

#### Data management and analysis

To ensure accurate statistical analysis, we applied weightings to the data based on sampling weight, primary sampling unit, and strata. This was done to restore the survey’s representativeness and account for the sampling design when computing standard errors. The aim was to obtain reliable statistical estimates. To manage and analyze our data, we used STATA version 17 statistical software. Given the study design and prevalence of breast cancer screening (>10%), multilevel modified Poisson regression with robust variance was fitted.

We preferred this model because of the following reasons. First, when the magnitude of the outcome variable is common, the odds ratio obtained using the binary logistic regression approach overestimates the strength of the relationship. Second, because the DHS data is hierarchical, women were nested within cluster/EA. As a result, our model considers data dependencies as well as the problem of overestimation.

The Likelihood Ratio (LR) test and intra-class Correlation Coefficient (ICC), were computed to measure the variation between clusters. The ICC quantifies the degree of heterogeneity between clusters.

ICC = ϭ^2^/ (ϭ^2^+π^2^/3) [20], *σ*^2^ indicates that cluster variance.

We have fitted four models separately. null model was fitted without independent variables to estimate the cluster-level variation of breast cancer screening in SSA. Model I and Model II were adjusted for individual-level variables and community-level variables, respectively. Model III was the final model adjusted for individual and community-level variables simultaneously. Variables with a p-value<0.2 in the bi-variable multilevel robust Poisson regression analysis were considered for the multivariable analysis. Deviance was used to verify model fitness and a model with the lowest deviance was considered the best-fit model. Finally, the Adjusted Prevalence Ratio (APR) with its 95% confidence interval (CI) was reported, and variables with a p-value <0.05 in the multivariable analysis were considered statistically significant.

### Ethical consideration

Hence, this study used secondary (DHS) data; it did not require ethical approval or participant consent. We have obtained permission to download and use the data from http://www.dhsprogram.com for this study. There are no names or addresses of individuals or households recorded in the datasets.

## Result

### Socio-demographic and health-related characteristics of the study participants

This study included 80,058 women of reproductive age from six SSA countries. Among the participants, 29.7%, 35.9%, and 7.6% had primary, secondary, and higher education, respectively. More than half of the participants were married (58.9%), and 56.8% resided in rural areas. The vast majority (78.2%) of participants had media exposure. While 63.9% of participants did not use contraceptives, 3.1% and 33.0% used traditional and modern contraceptives, respectively (Table 1).

**Table 1:** Socio-demographic and health-related characteristics of the study participants.

Variable	Weighted frequency	Breast cancer screening	
		**No (%)**	**Yes (%)**
**Community level variables**			
**Residence**			
Urban	34,597 (43.22)	27,453 (79.35)	7,144 (20.56)
Rural	45,461 (56.78)	39,839 (87.63)	5,622 (12.37)
**Distance to a health facility**			
Big problem	11,037 (13.79)	9,429 (85.43)	1,608 (14.57)
Not a big problem	69,021 (86.21)	57,863 (83.83)	11,158 (16.17)
**Individual level variables**			
**Age**			
15–19	16,524 (20.64)	15,365 (92.98)	1,159 (7.02)
20–24	14,825 (18.52)	12,736 (85.91)	2,089 (14.09)
25–29	12,997 (16.23)	10,627 (81.76)	2,370 (18.24)
30–34	11,481 (14.34)	9,247 (80.54)	2,234 (19.46)
35–39	10,204 (12.75)	8,141 (79.78)	2,063 (20.22)
40–44	7,899 (9.87)	6,244 (79.05)	1,655 (20.95)
45–49	6,127 (7.65)	4,933 (80.51)	1,194 (19.49)
**Educational status**			
No formal education	21,438 (26.78)	17,982 (83.88)	3,456 (16.12)
Primary	23,755 (29.67)	21,158 (89.07)	2,597 (10.93)
Secondary	28,749 (35.91)	23,949 (83.31)	4,799 (16.69)
Higher	6,116 (7.64)	4,202 (68.70)	1,914 (31.30)
**Marital status**			
Never in union	26,052 (32.54)	23,161 (88.90)	2,891 (11.10)
Currently married/ living with a man	47,211 (58.97)	38,422 (81.38)	8,789 (18.62)
Formerly married/ living with a man	6,795 (8.49)	5,709 (84.02)	1,085 (15.98)
**Wealth index**			
Poor	26,977 (33.70)	24,162 (89.57)	2,814 (10.43)
Middle	15,236 (19.03)	13,059 (85.71)	2,177 (14.29)
Rich	37,845 (47.27)	30,070 (79.46)	7,775 (20.54)
**Sex of household head**			
Male	56,169 (70.16)	47,164 (83.97)	9,004 (16.03)
Female	23,889 (29.84)	20,128 (84.26)	3,761 (15.74)
**Media exposure (n = 80,030)**			
No	17,474 (21.83)	15,671 (89.68)	1,803 (10.32)
Yes	62,556 (78.17)	51,597 (82.48)	10,959 (17.52)
**Parity**			
None	22,628 (28.26)	20,552 (90.82)	2,076 (9.18)
1–4	43,087 (53.82)	34,747 (80.64)	8,340 (19.36)
>4	14,343 (17.92)	11,993 (83.62)	2,350 (16.38)
**Contraceptive use**			
No	51,127 (63.86)	44,089 (86.23)	7,038 (13.77)
Traditional	2,487 (3.11)	2,001 (80.47)	486 (19.53)
Modern	26,444 (33.03)	21,201 (80.17)	5,242 (19.83)
**Pregnancy status**			
No/unsure	74,316 (92.83)	62,730 (84.41)	11,585 (15.59)
Yes	5,742 (7.17)	4,561 (79.43)	1,181 (20.57)
**Breastfeeding status**			
No	64,069 (80.03)	54,303 (84.76)	9,766 (15.24)
Yes	15,989 (19.97)	12,989 (81.24)	2,999 (18.76)
**Visited health facility last 12 months** **(n = 80,056)**			
No	35,315 (44.11)	31,586 (89.44)	3,729 (10.56)
Yes	44,741 (55.89)	35,705 (79.80)	9,036 (20.20)

### Prevalence of breast cancer screening among women of reproductive age in sub-Saharan Africa

**Fig 1:**
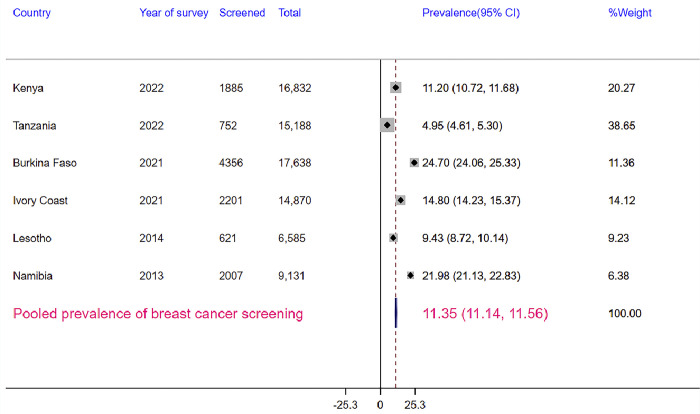
The pooled prevalence of breast cancer screening among women of reproductive age in sub-Saharan Africa.

The pooled prevalence of breast cancer screening in SSA was 11.35% (95% CI: 11.14%, 11.56%). The prevalence has varied across countries ranging from 4.95% (95% CI: 4.61%, 5.30%) in Tanzania to 24.70% (95% CI: 24.06%, 25.33%) in Burkina Faso (Fig 1).

### Statistical analysis and model comparison

Even though, the ICC value was less than 10% the Log-likelihood Ratio (LR) was significant, indicating that a multilevel binary logistic regression model better fits the data than the classical regressions. The Log-likelihood ratio test which was (X^2^  =  814.71, *p*-value < 0.001) informed us to choose the generalized linear mixed-effect model (GLMM) over the basic model. The models were compared with deviance and the final model with both individual and community-level variables was chosen as the best-fitted model since it had the lowest deviance value (58,161.96) (Table 2).

**Table 2:** Model comparison and random effect results.

	Null model	Model I	Model II	Model III
LR test	X^2^ = 814.71, *p-value<0*.*001*			
ICC %	6.00 (5.00, 7.00)	6.00 (5.00, 7.00)	5.00 (4.00, 6.00)	5.00 (4.00, 6.00)
Log likelihood ratio	-33138.01	-31076.27	-31144.55	-29080.98
Deviance	66,276.02	62,152.54‬	62,289.1‬	58,161.96‬
AIC	66280.02	62194.53	62307.1	58217.97
BIC	66298.6	62389.67	62390.74	58478.16

**Abbreviations:** AIC: Akaike’s information criterion, BIC: Bayesian information criterion; ICC: Intra-class Correlation Coefficient; LR test: Likelihood ratio test

### Factors associated with breast cancer screening among reproductive-age women in sub-Saharan Africa

In the multilevel binary logistic regression model, place of residence, age, educational status, media exposure, parity, contraceptive use, pregnancy status, breastfeeding, and visiting a healthcare facility in the last 12 months were statistically significant predictors of breast cancer screening.

Compared to women residing in urban residencies women from rural residencies had a prevalence of breast cancer screening decreased by 23% (APR = 0.77, 95%CI: 0.72, 0.83). The prevalence of breast cancer screening among women whose age is 20–24, 25–29, 30–34, and 35–39 was 30% (APR = 1.30, 95%CI: 1.19, 1.42), 41% (APR = 1.41, 95%CI: 1.29, 1.55), 61% (APR = 1.41, 95%CI: 1.29, 1.55), 84% (APR = 1.84, 95%CI: 1.67, 2.03) higher compared to women whose age is 15–19, respectively. The prevalence of breast cancer screening among women whose age is 40–44 and 45–49 were 2.06 times (APR = 2.06, 95%CI: 1.86, 2.28) and 2.19 times (APR = 2.19, 95%CI: 1.97, 2.43) higher, respectively. While having primary education decreases the prevalence of breast cancer screening by 30% (APR = 0.70, 95% CI: 0.65, 0.74), having secondary and higher education is found to increase the prevalence of breast cancer screening by 7% (APR = 1.07, 95% CI: 1.01, 1.14) and 48% (APR = 1.48, 95% CI: 1.37, 1.59), respectively, compared to those with no formal education. Compared to women to do not had media exposure women who had media exposure had 1.24 times (APR = 1.24, 95%CI: 1.17, 1.33) higher prevalence of breast cancer screening. Being multiparous and grand-multiparous increases the prevalence of breast cancer screening by 1.51 times (APR = 1.51, 95%CI: 1.41, 1.63) and 1.33 times (APR = 1.33, 95%CI: 1.21, 1.46) than being nulliparous. Women in middle and rich households had 1.26 times (APR = 1.26, 95%CI: 1.18, 1.35) and 1.43 times (APR = 1.43, 95%CI: 1.33, 1.54) higher prevalence of breast cancer screening compared to women from poor households. Compared to their counterparts’ women who were pregnant, breastfeeding, and visited healthcare facilities in the last 12 months had 1.56 times (APR = 1.56, 95%CI: 1.46, 1.66) 1.24 times (APR = 1.24, 95%CI: 1.18, 1.29) and 1.51 times (APR = 1.51, 95%CI: 1.44, 1.58) higher prevalence of breast cancer screening, respectively. Compared to women who do not use any time of contraceptives women who use traditional and modern contraceptives were found to have 1.16 times (APR = 1.16, 95%CI: 1.06, 1.27) and 1.28 times (APR = 1.28, 95%CI: 1.23, 1.33) higher prevalence of breast cancer screening (Table 3).

**Table 3:** Factors associated with breast cancer screening among sub-Saharan women of reproductive age.

Variable	Model I APR (95%CI)	Model II APR (95%CI)	Model III APR (95%CI)
**Community level variables**			
**Residence**			
Urban		1	1
Rural		0.62 (0.58, 0.67)	0.77 (0.72, 0.83) *
**Distance to a health facility**			
Not a big problem		1	1
big problem		0.92 (0.87, 0.98)	1.02 (0.97, 1.08)
**Individual level variables**			
**Age**			
15–19			1
20–24	1.31 (1.21, 1.43)		1.30 (1.19, 1.42) *
25–29	1.43 (1.31, 1.57)		1.41 (1.29, 1.55) *
30–34	1.63 (1.48, 1.79)		1.61 (1.46, 1.76) *
35–39	1.86 (1.69, 2.06)		1.84 (1.67, 2.03) *
40–44	2.09 (1.88, 2.32)		2.06 (1.86, 2.28) *
45–49	2.20 (1.97, 2.45)		2.19 (1.97, 2.43) *
**Educational status**			
No formal education	1		1
Primary	0.70 (0.65, 0.74)		0.70 (0.65, 0.74) *
Secondary	1.07 (1.01, 1.14)		1.07 (1.01, 1.14) *
Higher	1.49 (1.38, 1.60)		1.48 (1.37, 1.59) *
**Marital status**			
Never in union	1		1
Currently married/ living with a man	1.04 (0.98, 1.11)		1.05 (0.99, 1.12)
Formerly married/ living with a man	0.93 (0.86, 1.01)		0.94 (0.86, 1.01)
**Wealth index**			
Poor	1		1
Middle	1.32 (1.24, 1.41)		1.26 (1.18, 1.35) *
Rich	1.64 (1.53, 1.75)		1.43 (1.33, 1.54) *
**Sex of household head**			
Male	1		1
Female	1.02 (0.97, 1.06)		1.00 (0.96, 1.05)
**Media exposure**			
No	1		1
Yes	1.25 (1.17, 1.33)		1.24 (1.17, 1.33) *
**Parity**			
Nulliparous	1		1
Multiparous	1.51 (1.40, 1.63)		1.51 (1.41, 1.63) *
Grand multiparous	1.31 (1.19, 1.43)		1.33 (1.21, 1.46) *
**Contraceptive use**			
No	1		1
Traditional	1.16 (1.05, 1.27)		1.16 (1.06, 1.27) *
Modern	1.28 (1.23, 1.33)		1.28 (1.23, 1.33) *
**Pregnancy status**			
No/unsure	1		1
Yes	1.56 (1.46, 1.66)		1.56 (1.46, 1.66) *
**Breastfeeding status**			
No	1		1
Yes	1.23 (1.18, 1.29)		1.24 (1.18, 1.29) *
**Visited health facility last 12 months**			
No	1		1
Yes	1.50 (1.43, 1.57)		1.51 (1.44, 1.58) *

Notes

*significant in adjusted regression analysis, 1 = Reference

**Abbreviations:** APR: adjusted prevalence ratio; CI: confidence interval

## Discussion

In this study, the pooled prevalence of breast cancer screening among reproductive-age women in SSA was 11.35%, (95% CI: 11.14%, 11.56%). This finding is lower than with findings from previous studies [17, 19]. This could stem from variations in healthcare infrastructure among the study areas. Additionally, it might be attributed to constrained resources for breast cancer screening and the inadequacy of healthcare facilities to provide sufficient breast cancer screening services in countries across SSA.

The multilevel robust Poisson regression analysis revealed that various factors influence breast cancer screening. Accordingly, corroborated with previously done studies in Africa [17, 19], compared to urban residents women who reside in rural were found to have a low prevalence of breast cancer screening. In rural Africa; accessing healthcare is a major challenge due to several factors such as socioeconomic determinants of health, transportation-related obstacles, and urban bias [21]. Consequently, women living in rural areas have a significantly lower chance of undergoing breast cancer screening.

Consistent with previous studies [22, 23], compared to younger women older women were more likely to utilize breast cancer screening services. The association between older age and undergoing breast cancer screening may be attributed to the heightened awareness among older women regarding the benefits of early detection in mitigating the risk of developing breast cancer. Additionally, older women are increasingly susceptible to breast cancer, in comparison to their younger counterparts, thereby elevating the likelihood of contracting the disease.

In line with previous studies done in low-resource countries [24], women who had higher levels of educational status were found to have high tendencies to utilize breast cancer screening, while those with only primary education had lower tendencies to do so, compared to women with no formal education. Women who have received higher education are often more knowledgeable about health complications and the potential negative effects of diseases. They tend to prioritize their health by seeking out reproductive health check-ups, screening services for diseases like breast and cervical cancer, and utilizing prevention strategies such as vaccinations and additional screening services. As a result, they are more likely to make use of breast cancer screening services.

This study found that women with middle and richer wealth indexes were more likely to undergo breast cancer screening in contrast to poorer women. This aligns with conclusions drawn in earlier studies [17, 19, 25]. This might be because women endowed with significant financial resources demonstrate a greater propensity to avail themselves of preventive healthcare services, including breast cancer screening. On the contrary, those facing economic challenges might deprioritize such preventive care activities, prioritizing essential needs like providing sustenance for their families.

Similar to previously done studies [26, 27], the current study found a significant association between household media exposure and increased utilization of breast cancer screening services. Research has unequivocally established that media exposure is the most effective and influential method for elevating awareness of breast cancer screening and early detection strategies, including breast screening or examination-related services [28]. Women who have been exposed to media are more likely to possess knowledge about breast health and cancer screening strategies, thereby increasing the likelihood of their utilization of breast cancer screening services. In light of this finding, it is important for healthcare providers to consider the potential impact of media exposure on women’s health knowledge, and to take advantage of media outlets as a means of disseminating accurate and timely information about breast cancer screening. This approach may help to increase awareness and participation in breast cancer screening programs, ultimately leading to improved health outcomes for women.

Our analysis also revealed that being multiparous and grand-multiparous increases the prevalence of breast cancer screening compared to being nulliparous. The possible justification could be that women who have experienced multiple pregnancies might possess a heightened awareness of their bodily changes, particularly in their breasts. This awareness, coupled with the education they may receive during prenatal visits, could increase the chance of undergoing breast cancer screenings. Moreover, these women may have more regular interactions with healthcare professionals, creating additional opportunities for providers to recommend and conduct breast cancer screenings. Conversely, another study indicated that those with higher parity were less likely to be screened for breast cancer [19].

According to the current study, contraceptive use was associated with an increased prevalence of breast cancer screening among women of reproductive age. The possible explanation could be women who use contraceptives tend to frequently visit healthcare facilities and have knowledge about reproductive health-related health problems [29], and many healthcare providers require women to undergo routine screening [30]. Furthermore, even though the risk is very low, hormonal contraceptives have been linked to an increased risk of breast cancer [31–33], so when women develop lumps or other symptoms on their breasts, they are more likely to get breast cancer screening.

Furthermore, this study highlighted the association between pregnancy and breastfeeding status with breast cancer screening uptake. Accordingly, compared to their counterparts, women who were pregnant showed a higher prevalence of breast cancer screening. This finding aligns with the results of a study conducted in Africa [17]. Similarly, women who breastfed exhibited a higher prevalence of breast cancer screening. Consistent with a previous study [17], this study also found that women who had visited healthcare facilities in the past 12 months demonstrated a higher likelihood of undergoing breast cancer screening.

## Strength and limitations

The strength of the study lies in its use of nationally representative data, providing findings that are applicable across the sampled countries. The study’s robustness is enhanced by the employment of a larger sample size and advanced statistical methods. However, establishing causality between dependent and independent variables is impossible due to the cross-sectional nature of the study.

## Conclusion

Despite the high mortality rates associated with breast cancer in SSA, this study has revealed a low prevalence of breast cancer screening, highlighting significant disparities across different countries in the region. Place of residence, age, educational status, media exposure, parity, contraceptive use, pregnancy status, breastfeeding, and visiting a healthcare facility in the last 12 months were found to be significantly associated with breast cancer screening. The study results emphasize the importance of interventions focused on encouraging breast cancer screening behaviors in this region. Recognizing the challenges faced by many women, there is a crucial need to promote education and awareness about the advantages of undergoing breast cancer screening.
